# Dual-harm in adolescence and associated clinical and parenting factors

**DOI:** 10.1007/s00127-022-02258-2

**Published:** 2022-03-29

**Authors:** Pascalle Spaan, Philip J. S. Michielsen, Nita G. M. de Neve-Enthoven, Diandra C. Bouter, Nina H. Grootendorst-van Mil, Witte J. G. Hoogendijk, Sabine J. Roza

**Affiliations:** 1 Department of Psychiatry, Erasmus MC University Medical Center, Dr. Molewaterplein 40, 3015 GE Rotterdam, The Netherlands; 2 Mental Health Institute, GGZ Westelijk Noord-Brabant, Halsteren, The Netherlands; 3 Epidemiological and Social Psychiatric Research Institute (ESPRi), Department of Psychiatry, Erasmus MC University Medical Center, Rotterdam, The Netherlands; 4 Netherlands Institute for Forensic Psychiatry and Psychology, The Hague, The Netherlands

**Keywords:** Dual-harm, Adolescence, Aggression, Violence, Self-injury

## Abstract

**Purpose:**

Both aggression toward others and self peak in adolescence and interpersonal violence and suicide are among the leading causes of death in young people worldwide. Individuals who show both aggression toward others and self, i.e. *dual-harm*, may experience the worst outcomes. The current study investigates clinical and parenting factors associated with dual-harming in adolescence, to provide new insights for prevention and treatment.

**Methods:**

In a prospective cohort of adolescents, oversampled on emotional and behavioral problems (*n* = 1022; aged 12–17 years), we investigated co-occurrence in harm toward others and self and presented findings in an area-proportional Euler diagram. Four harm groups (no harm, other-harm, self-harm, and dual-harm) were compared on intelligence scores, general functioning, emotional and behavioral problems, substance use, parental hostility, and harsh parenting with ANCOVAs and logistic regressions.

**Results:**

In adolescents that other-harmed, the risk of self-harm was 1.9 times higher than for those who did not harm others. Dual-harm adolescents reported worse overall functioning, more emotional and behavioral problems, more parental hostility and harshness, and were more likely to use substances than those who did not engage in aggressive behaviors. No evidence of differences in intelligence scores between groups were found.

**Conclusion:**

These findings highlight a vulnerable group of adolescents, at risk of future suicide, violent offending, and the development of severe psychopathology. Dual-harm is a promising marker for early intervention and referral to specialized mental health professionals. Further research is needed to examine underlying pathways and risk factors associated with persistent dual-harm trajectories into adulthood.

**Supplementary Information:**

The online version contains supplementary material available at 10.1007/s00127-022-02258-2.

## Introduction

Antisocial behavior and self-harm are considered major public health problems that, in addition to burdening individuals and families, inflict considerable costs on society [1, 2]. Both aggression toward others and toward self peak in adolescence [3, 4]. Aggression toward others can include verbal, psychological, or physical aggression, as well as violent crime. The prevalence of violent offending peaks around age 15–19 years [5]. Aggression toward self can take the form of suicidality or (non-suicidal) self-injury (NSSI). About a quarter of adolescents (12–17 years) experience suicidal ideation [6] and completed suicide is the third main cause of death in teenagers worldwide [7]. NSSI, where body tissue is destroyed deliberately by self-infliction and without suicidal intent, has a typical age of onset between 12 and 14 years [6, 8]. When two types of behavior are prevalent in adolescence, their co-occurrence within individuals is, in itself, not surprising. However, co-occurring aggression toward others and self may be more prevalent than expected on the basis of chance. This multimorbidity may point to higher severity of problems and worse prognosis throughout life than other-harm or self-harm alone, and thus mark a specific subgroup of interest for early prevention and intervention.

Recently, some studies have focused on *dual-harm*, i.e. the combined occurrence of aggression toward others and aggression toward self [9, 10]. A systematic review by O’Donnell et al. [9] describes that studies usually show an increased rate of aggression or violence toward others in adolescents with a history of NSSI (*k* = 1; OR = 9.71; *N* = 33) and suicidal behavior (*k* = 10; ORs range from 0.79 to 11.14; *N* = 456). However, literature on adolescent dual-harm so far is scarce. A study indicates that late adolescents (around 18 years old) who dual-harm, have more psychological risk factors and problems than self-only harmers, such as a higher likelihood of child victimization, lower self-control, and childhood IQ, and more substance use problems, psychotic symptoms, resistance to change, and emotional regulation problems [11]. Despite these problems, dual-harming adolescents are not more likely to receive mental healthcare. Moreover, a population-based study indicates that dual-harming individuals aged 15–35 years old are at greater risk of dying from unnatural causes, like suicide or accidents, than those who self-harm only and those who other-harm only [12]. In terms of mental illness, a developmental trajectory that contains both aggression toward others and self can be indicative of increased risk for DSM-5 disorders [13], such as substance use disorders [14], antisocial personality disorder, and borderline personality disorder [13]. Both aggression toward others and self are associated with (preclinical) symptoms of borderline personality disorder, such as impulsivity, intense anger, avoidance of abandonment and feelings of emptiness [15]. These findings indicate that the combination of aggression toward others and self may mark more severe psychopathology and a worse prognosis than either behavior separately.

Clustering of two types of behavior can be expected when they are causally related or due to the presence of common underlying risk factors. One possible explanation of the co-occurrence between aggressive behavior, violent offending, suicidality and NSSI in adolescence is shared biological underpinnings, e.g. (epi)genetic deficits in serotonin and dopamine pathways [16]. In terms of parental psychopathology, antisocial personality disorder and attempted suicide in one or both parents are strong predictors of violent offending in adolescent offspring [17]. Furthermore, parental antisocial personality disorder and mood disorders increase the lifetime risk of offspring suicidality, specifically during the vulnerable adolescent stage [18]. Beyond psychopathology in one or both parents, parental hostility and harsh parenting may be risk factors for aggression toward others and self in offspring. Hostility is a persistent feeling of anger covering emotional, cognitive, and behavioral aspects [19]. Parental hostility is a stronger predictor for aggression and conduct problems in young children than parental depression [19]. Furthermore, parental hostility partially explains the association between maternal antisocial behavior and symptoms of disruptive behavior disorder in offspring [20]. However, the relationship between parental hostility and child aggressive behavior is likely bidirectional: aggressive behavior in adolescents can both precede and be the result of parental hostility [21]. Harsh parenting involves aversive parenting behaviors, such as physical and verbal punishment, and is associated with offspring physical aggression and suicidal ideation in adolescence [22]. Although both parental hostility and harshness have been related to adolescents’ aggression toward others; it remains unknown whether these associations are stronger in individuals who dual-harm.

In the current study, we aim to investigate the co-occurrence of harm toward others and self in a large community-based group of adolescents, oversampled on their risk of emotional and behavioral problems. The current study considers broad definitions in which aggression toward others includes physical aggressive behavior toward others as well as violent offending and aggression toward self includes NSSI, recent suicidal ideation, and suicide attempts. Dual-harm is a concept for which the definition and boundaries still are in need of further study [23], therefore we chose to include a broad group of behaviors, including various forms of aggression toward others or self. To account for contextual variations and (self-)reporter biases, we use a multi-informant approach to assess harming behaviors. We aim to compare adolescents with aggression toward both others and self, aggression toward others only, and aggression toward self only, to adolescents without any aggressive behavior on various biopsychosocial clinical and parenting factors. We expect that adolescents that dual-harm report the lower intelligence scores and general functioning, and more emotional and behavioral problems, substance use, parental hostility and harshness than no-harm adolescents. These factors, namely intelligence scores, general functioning, emotional and behavioral problems, substance use, parental hostility and harshness, may provide valuable targets for prevention and intervention efforts. Furthermore, findings from the current study may yield new insights in prevention of complex later-life psychopathology such as persistent (violent) offending, completed suicide, and personality disorders.

## Method

### Study design

The current study used data from the iBerry Study, a prospective population-based cohort study on the development of adolescent subclinical psychiatric symptoms into psychiatric disorders; the study design is described in detail by Grootendorst-van Mil and Bouter et al. [24]. In a larger area (including rural and urban regions) in the Netherlands, children in the first year of secondary school between 2014 and 2016 completed the Strengths and Difficulties Questionnaire-Youth (SDQ-Y) [25]. From these 16,736 adolescents aged 12–15 years, a selection was made based on their SDQ-Y score, stratified by sex. All high-risk adolescents (top 15% scores) and a random sample of low-risk adolescents (lowest 85% scores) were selected, with a 2.5:1 ratio between the number of high-risk and low-risk adolescents to create a cohort with sufficient power to study less common outcomes. At baseline, 1022 adolescents participated (54% response rate) by visiting the research center between September 2015 and 2019, usually accompanied by a parent or primary caregiver. Adolescents and (accompanying) parents or caregivers provided informed consent and completed interviews, questionnaires, and biological measurements. Adolescents received a small monetary compensation. Researchers were blind to screening status. The Erasmus Medical Center’s Medical Ethics Review Committee approved the study protocol (MEC-2015-007).

### Measurement of aggression toward others

#### Aggressive behavior

The Youth Self-Report (YSR 11–18) of the ASEBA system was used to measure adolescents’ self-reported aggressive behavior over the past 6 months [26]. After exclusion of emotional regulation problems, positive answers on the items *gets into fights*, *attacks people*, *teases a lot*, and *threatens others* were considered as recent aggressive behavior.

The MINI-KID is a semi-structured clinical interview to determine psychopathology in adolescents, classified according to 23 DSM-IV categories [27]. We used items from the conduct disorder module (P). Positive answers to questions regarding *bullying or threatening others *and *firesetting with intent to damage* in the past year were considered as recent aggressive behavior.

#### Violent offending

A Dutch adaptation of the Self-Reported Early Delinquency (SRED) was used to measure violent offending in the past 6 months [28]. This interview consisted of 23 items. Six items were considered violent based on the Statistics Netherlands classification [29]: *joining a fight, hitting someone in public, carrying a weapon, hitting someone resulting in use of medical care, robbery*, and *fighting with a weapon*. Any positive answer on these items was considered recent violent offending.

The conduct disorder module of the MINI-KID was also used for information on violent offending in the past year. Any positive answer to the questions regarding *starting fights, using a weapon, intentionally hurting a person, intentionally hurting an animal, robbery*, and *forcing sexual contact* was considered recent violent offending.

### Measurement of aggression toward self

#### Suicidality

The VOZZ-SCREEN, a Dutch self-report screener of suicidal risk, was used to determine lifetime suicidality [30]. It consists of ten items with a 5 point Likert scale. Lifetime presence was scored dichotomously based on at least one positive answer on items 8–10, which regard recent suicidal thoughts (past 7 days) and lifetime attempts, or on item 91 of the YSR considering suicidal thoughts in the past 6 months.

#### NSSI

The self-report Inventory of Statements about Self-Injury (ISAS) was used to measure lifetime NSSI [31]. Participants reported how often they engaged in self-harm with the intention of hurting oneself, for example by *cutting, biting, *and *banging or hitting oneself*. Lifetime presence was scored dichotomously based on any self-reported NSSI form except non-intentional *interfering with wound healing, swallowing dangerous substances*, and *other*.

### Measurement of harm groups

Harm groups were categorized based on non-imputed data. Adolescents who scored on both other-harm (aggressive or violent behavior in the past 6 months) and self-harm (lifetime suicidality or NSSI) were categorized in the dual-harm group. The adolescents who only harmed others, only harmed themselves, or those who did neither were categorized in, respectively, the other-harm group, self-harm group, and no-harm group.

### Measurement of clinical factors

#### Intelligence scores

The SON-R 6-40 is a non-verbal intelligence test for individuals aged 6–40 years, which is relatively insensitive to cultural differences [32]. The subtests analogies and categories were conducted; both measure general reasoning skills. These subtest scores correlate strongly with total intelligence scores (0.68 and 0.59, respectively).

#### General functioning

The Child Outcome Rating Scale (CORS) is a short assessment scale filled out separately by the adolescent, parent, and clinician who interviewed the adolescent to measure functioning of the adolescent in four domains: individual, at home, at school, and overall [33]. It consists of four visual analogue scales from 0 to 10. Scores across domains are added for a general functioning score; higher scores indicate better functioning.

#### Total emotional and behavioral problems

Parents reported on their children’s emotional and behavioral problems in the past 6 months on the Child Behavior Checklist (CBCL 6–18) [26]. Higher scores indicated more emotional and behavioral problems.

#### Substance use

Adolescents reported on their substance use in the SRED and MINI-KID interviews, in the YSR questionnaire and as part of a self-constructed questionnaire. Answers to the interviews and questionnaires were combined and dichotomized to indicate either presence or absence of lifetime alcohol use, smoking, or illicit drug use.

### Measurement of parenting factors

#### Parental hostility

The Dutch version of the Brief Symptom Inventory (BSI) was used to measure parental hostility; symptoms were self-reported by the parent over the past 7 days [34]. The hostility scale contains five items which can be answered on a 3-point Likert scale from *not at all or little* (0) to *often* (2). Higher scores indicate higher levels of hostility.

#### Harsh parenting

The 10-item version of the Parent–Child Conflict Tactic Scale (CTS-PC) was used to measure harsh parenting in the past 2 weeks [35]. Answers are scored on a 3-point Likert scale from *never* (0) to *often* (2). The adolescent reported on the harsh parenting of both parents, if applicable, and these scores were averaged. Additionally, the accompanying parent reported on their harsh parenting regarding the adolescent. For both informants a sum score was calculated from the psychological aggression (e.g. shouting) and physical assault (e.g. shaking) subscales, with higher scores indicating harsher parenting.

### Measurement of demographics

Educational level of the adolescent was coded as intermediate (pre-vocational education), intermediate/high (secondary general education), higher (pre-university education), or mixed (decision between two educational levels comes at a later stage). Educational level of the parent was based on the highest obtained diploma as either lower (primary school or secondary pre-vocational training; corresponding to less than 12 years of education), intermediate (vocational training, secondary general or pre-university education; corresponding to about 13–15 years of education), higher (higher or academic education; corresponding to over 16 years of education), or other. Adolescents’ ethnic background was coded based on the country of birth of one's parents as Dutch, other Western, or non-Western.

### Statistical approach

First, the co-occurrence in harm toward others and self was studied by means of a Chi^2^ test and presented in an area-proportional Euler diagram; the diagram was made with the Eulerr package in R. Next, the four harm groups (no harm, other-harm, self-harm, and dual-harm) were compared by ANCOVA on their intelligence scores, general functioning, emotional and behavioral problems, parental hostility, and harsh parenting scores. Sidak corrections were used for multiple pairwise comparisons of estimated means. Groups were compared on substance use in a logistic regression model. Models were adjusted for parental age, sex, education and adolescents' age and sex. Sensitivity analyses were conducted to study the impact of operationalizing other-harm as aggressive behavior only and a second set operationalizing self-harm as suicidality only to explore the potential influence of different dual-harm operationalizations. Results of unadjusted analyses and sensitivity analyses are presented in Online Resource 1, Tables S1–S6. SPSS V.25 (IBM Corp., Armonk, NY) was used for all analyses and multiple imputation. A *p *value below 0.05 was considered statistically significant.

### Missing data and multiple imputation

Missing data resulted primarily from declined interviews or unreturned questionnaires. The amount of missing data differed per instrument: suicidality (0%); demographics data (0.0–11.7%); substance use (< 1%); aggressive behavior, violent offending, NSSI, and intelligence scores (5.1–5.5%); parent-reported emotional and behavioral problems, parental hostility, and harsh parenting (12.5–18.2%); and general functioning (35.7–59.3%). The missing values on general functioning were higher because the instrument was added after about a third of the sample was included.

For sum scores, when 75% of the items were valid, the average item score on valid items was multiplied by the number of items in the scale to estimate scores. Missing values after these calculations were assumed to be missing at random and handled by multiple imputation. Five imputed datasets were created under fully conditional specification (FCS) with 1000 iterations per chain. Scale variables were imputed with predictive mean matching (PMM) and binary variables with logistic regression. Auxiliary variables were used (SDQ-Y screening status, Prodromal Questionnaire-16 psychotic experiences sum scores, YSR externalizing and internalizing scores). Plots showed sufficient convergence. Coefficients from ANCOVA and logistic regression were pooled across imputation sets, based on Rubin’s Rules that take into account within and between imputation variance [36]. For statistics not pooled automatically, median values were reported [37].

## Results

### Sample

Table 1 shows demographic characteristics by sex and harm group based on the original, non-imputed, data of 1022 adolescents. A similar number of boys and girls participated, on average 15 years old, and most were in intermediate (pre-vocational) education. In total, 19.9% of adolescents reported aggressive behavior, 35.7% reported violent offending, 12.3% reported suicidality, and 33.3% reported NSSI. This resulted in four groups: no harm (*n* = 437), other-harm (*n* = 235), self-harm (*n* = 175), and dual-harm (*n* = 175). Accompanying parents were most often female (82.9%), on average 47 years old and intermediate to higher educated.

**Table 1:** Descriptive characteristics of the adolescent sample by sex and harm group

Characteristic (*n*, %)^a^	Missing%	Total		Boys		Girls		No harm		Other harm		Self-harm		Dual harm	
		*n* = 1022		*n* = 500		*n* = 522		*n* = 437		*n* = 235		*n* = 175		*n* = 175	
Adolescent															
Age (*M*, SD)	0	15.0	0.9	15.0	0.9	15.0	1.0	15.0	1.0	15.0	0.8	15.0	1.0	15.0	0.9
Sex															
Female	0	522	51%	–	–	522	100%	243	56%	64	27%	129	74%	86	49%
Education															
Intermediate	6	467	49%	223	47%	244	50%	196	46%	111	52%	81	49%	79	51%
Intermediate/high		219	23%	100	21%	119	24%	102	24%	45	21%	34	20%	38	25%
Higher		185	19%	94	20%	91	19%	89	21%	36	17%	34	20%	26	17%
Mixed		88	9%	54	11%	34	7%	37	9%	23	11%	17	10%	11	7%
Ethnic background															
Dutch	10	709	77%	345	77%	364	78%	317	80%	155	74%	128	81%	109	72%
Other Western		55	6%	26	6%	29	6%	21	5%	16	8%	7	4%	11	7%
Non-Western		151	17%	76	17%	75	16%	58	15%	39	19%	23	15%	31	21%
Parent															
Age (*M*, SD)	6	46.6	5.7	46.89	5.5	46.3	5.8	46.9	5.3	46.1	6.4	46.6	5.4	46.3	5.9
Sex															
Female	6	800	83%	369	78%	431	88%	350	83%	181	83%	136	83%	133	83%
Education															
Lower	11	173	19%	86	19%	87	19%	67	17%	46	22%	28	18%	32	21%
Intermediate		336	37%	167	37%	169	37%	140	35%	78	38%	61	39%	57	38%
Higher		303	33%	148	33%	155	34%	156	39%	57	28%	48	31%	42	28%
Other		95	10%	45	10%	50	11%	35	9%	23	11%	18	12%	19	13%

^a^Unless otherwise specified

Female adolescents reported less aggressive behavior and violent offending (respectively: *χ*^2^_1_ = 19.28, *p* < 0.001; *χ*^2^_1_ = 45.89, *p* < 0.001), and more lifetime suicidality and NSSI (respectively: *χ*^2^_1_ = 23.82, *p* < 0.001; *χ*^2^_1_ = 18.32, *p* < 0.001) than male adolescents. Females were somewhat overrepresented in the no-harm and self-harm groups, whereas males were overrepresented in the other-harm group (*χ*^2^_3_ = 93.20, *p* < 0.001). The dual-harm group’s sex distribution was as expected, approximately half-half.

### Co-occurrence of other-harm and self-harm

**Fig. 1:**
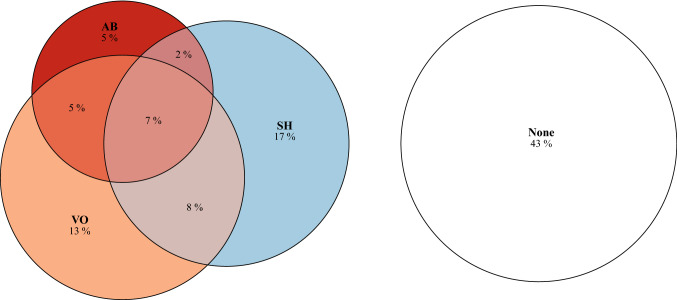
Area-proportional Euler diagram with co-occurrence of harming behaviors in adolescents (*n* = 1022). *AB* aggressive behavior, *SH* self-harm, *VO* violent offending

Other-harmers were almost twice as likely to self-harm and vice versa (OR 1.88, 95% CI 1.45–2.45). In adolescents who other-harmed, 42.7% reported lifetime self-harm with 15.9% reporting suicidality and 40.4% reporting NSSI. In adolescents who self-harmed, 50.3% reported harming others with 27.7% reporting aggression and 45.3% reporting violent offending. The co-occurrence between harming behaviors is shown in more detail in the area-proportional Euler diagram in Fig. 1. For example, the most reported co-occurring behaviors were violent offending combined with, respectively, NSSI and aggressive behavior (8% and 5%); 7% of adolescents reported all three of these behaviors.

### Associations of harm-groups with clinical and parenting factors

Table 2 shows the pooled pairwise comparisons of each harm-group regarding clinical and parenting factors, with the no harm group as reference. We found no evidence for differences between harm groups on intelligence scores. Regarding general functioning, adolescents in the dual-harm and self-harm groups rated their functioning the lowest (but did not differ statistically differ from one another), the other-harm group was the next lowest, and the no-harm group had the highest functioning scores (*η*_*p*_^2^ = 0.19). According to parents, the dual-harm group scored the lowest on general functioning, the other-harm group and self-harm group scored higher, and the no-harm group had the highest functioning scores (*η*_*p*_^2^ = 0.11). For clinician-rated functioning scores, the dual-harm group again scored the lowest, together with self-harm group, then the other-harm group, and the no harm group scored the highest (*η*_*p*_^2^ = 0.17). Dual-harm adolescents had the most total behavioral and emotional problems as rated by the parents, the other-harm and self-harm groups were next highest, and the no-harm group had the lowest problem scores of all groups (*η*_*p*_^2^ = 0.10). In short, the dual-harm group had the lowest functioning scores and highest problem scores in these comparisons.

**Table 2:** Results ANCOVA post hoc group comparisons by harm group, with no harm as reference group

Outcome	No harm		Other harm		Self-harm		Dual-harm		Between-subject effects^c^		No harm	
	Est. Mean	SE	Mean diff	SE	Mean diff	SE	Mean diff	SE	*F*_3,1011_	*p*	*η*_*p*_^2^	Effects
Clinical factors												
Intelligence scores^a^	98.43	0.66	− 2.80	1.12	− 0.26	1.22	− 1.68	1.21	2.37^a^	0.069	–	N,O,S,D
Functioning—adolescent rated^b^	33.26	0.27	− 2.21	0.54	− 4.84	0.63	− 6.50	0.52	80.05	< 0.001	0.18	N > O > S, D
Functioning—parent rated^b^	32.23	0.41	− 2.50	0.85	− 3.40	0.74	− 5.86	0.75	42.70	< 0.001	0.11	N > O, S > D
Functioning—clinician rated^b^	31.85	0.31	− 2.12	0.53	− 4.46	0.46	− 5.65	0.51	70.84	< 0.001	0.18	N > O > S, D
Total emotional and behavioral problems^b^	22.29	0.98	+ 8.93	1.72	+ 9.74	1.98	+ 17.90	1.96	37.70	< 0.001	0.10	N < O, S < D
Parenting factors												
Parental hostility^b^	0.68	0.06	+ 0.07	0.10	− 0.03	0.10	+ 0.29	0.10	4.25	0.005	0.01	N, S < D;O
Harsh parenting—adolescent rated^b^	1.39	0.09	+ 0.54	0.16	+ 0.63	0.18	+ 1.08	0.18	15.20	< 0.001	0.04	N < O < D; N < S
Harsh parenting—parent rated^b^	0.77	0.08	+ 0.38	0.17	+ 0.14	0.13	+ 0.74	0.16	13.92	< 0.001	0.04	N < O, D; S < D

*D* dual-harm, *N* no harm, *O* other harm, *S* self-harm

^a^Controlling for adolescents’ age and sex and parental age and sex; *F*_3,1014_

^b^Controlling for adolescents’ age and sex and parental age, sex, and education level

^c^Median statistic over pooled datasets

Parenting factors also differed between groups. First, parental hostility scores were higher in the dual-harm group than in the self-harm and no-harm groups (*η*_*p*_^2^ = 0.01). The other-harm groups’ score did not differ significantly from any other group. Second, dual-harm adolescents reported higher harsh parenting scores than the other-harm group, again with the no-harm group scoring lowest (*η*_*p*_^2^ = 0.04). The self-harm group reported more harsh parenting than the no harm group. Finally, parent-reported harsh parenting scores were higher in the dual-harm and other-harm group than in the no harm group (*η*_*p*_^2^ = 0.04). Furthermore, the dual-harm group reported higher harsh parenting scores than the self-harm group. To sum up, the dual-harm group reported the highest parental hostility and harsh parenting scores.

### Associations of harm-groups with substance use

In the total sample, 51% reported any substance use (18% smoking; 48% alcohol; 14% illicit drugs). The most frequently mentioned illicit drugs were cannabis (8%) and nitrogen oxide (3%). Table 3 shows the pooled results of covariate-adjusted logistic regression analyses regarding substance use, with the no harm group as reference. Adolescents in the dual-harm group were 8 times more likely to have smoked, around 3.5 times more likely to have used alcohol and 7 times more likely to have used illicit drugs compared to those in the no-harm group. The self-harm and other-harm groups also reported more substance use than the no harm group.

**Table 3:** Logistic regression results for harm groups on substance use

Outcome	Harm group (reference = no harm)										
	Other-harm			Self-harm			Dual-harm			Block 2 test^b^	
	OR	95% CI	*p*	OR	95% CI	*p*	OR	(95% CI)	*p*	*χ*^2^_3_	*p*
Smoking^a^	4.44	(2.71–7.29)	< 0.001	2.96	(1.74–5.04)	< 0.001	8.12	(4.99–13.23)	< 0.001	85.17	< 0.001
Alcohol^a^	2.36	(1.66–3.36)	< 0.001	1.59	(1.08–2.33)	0.019	3.53	(2.39–5.22)	< 0.001	50.04	< 0.001
Illicit Drugs^a^	4.42	(2.55–7.67)	< 0.001	2.54	(1.36–4.75)	0.003	7.01	(4.04–12.16)	< 0.001	59.03	< 0.001

^a^Controlling for adolescents’ age and sex and parental age, sex, and education level

^b^Median statistic over pooled datasets

With the dual-harm group as reference group, the other-harm reported significantly less smoking, and the self-harm group reported less smoking, alcohol and illicit drug use. The differences between dual-harm group and other-harm group in terms of alcohol use and illicit drug use did not reach significance (alcohol OR 0.67, 95% CI 0.44–1.03; illicit drugs OR 0.63, 95% CI 0.38–1.04). Furthermore, there was no evidence for differences between the self-harm and other-harm groups on substance use.

### Sensitivity analyses

In the current sample, the most prevalent behaviors in terms of other-harm and self-harm were, respectively, violent offending and NSSI. Therefore, these behaviors heavily influenced the classification in harm groups. A first set of sensitivity analyses was conducted with other-harm defined as aggressive behavior only and a second set with self-harm defined as suicidality only to explore the potential influence of the dual-harm operationalization. Results of the sensitivity analyses with regard to all explored factors, that is, clinical factors including substance use, and parenting factors, were consistent with the original analyses.

## Discussion

In this Dutch high-risk sample, other-harm and self-harm often co-occurred and dual-harm adolescents performed worse on several clinical factors, such as experiencing lower general functioning, more emotional and behavioral problems, and more substance use compared to no-harmers, but often also compared to other-only and self-only harmers. The dual-harm group also experienced parental hostility and harshness more often. No evidence was found for differences in intelligence scores between groups. This study adds to the knowledge on dual-harm in adolescence in several ways. First, it shows that dual-harming adolescents experience a broad range of biopsychosocial risk factors and problems that may ask for transdiagnostic approaches to prevention and treatment. Their lower general functioning and higher number of emotional and behavioral problems indicate their need for (mental) healthcare. In particular, the finding that dual-harmers experience more psychopathology, more risk factors and lower functioning even compared to those that other-harm or self-harm only, highlights the importance of considering dual-harmers as a unique high-risk group [23]. Unfortunately, earlier studies indicate that dual-harmers are not more likely to receive mental healthcare than self-only harmers [11]. It also demonstrates that broader categories of both other-harm (including various types of aggressive and violent behavior) and self-harm (including recent suicidality) in this sample resulted in similar outcomes as studies using narrower definitions, as further evidenced by sensitivity analysis results [9, 11, 12]. Future research may take into account that both aggression toward others and self are heterogeneous behaviors and may present in different severities [23]. More research is needed to establish whether the range and severity of dual-harm behavior influence the prognosis of adolescents. Whereas most studies in adolescents were conducted in clinical samples after a conviction or admission resulting from violent conduct or suicide attempts [9], this study identifies a vulnerable group likely at risk of future convictions or admissions for harming others or self. This is important from a prevention perspective, because detained dual-harming individuals can cause considerable strain on custodial or mental health care systems, for example by more frequently destroying property and setting fires and using more lethal self-harm methods than self-only or other-only harmers [38].

Second, compared to no-harmers and self-harmers, dual-harmers were more likely to smoke, use alcohol, and use illicit drugs. This is of concern, as there is substantial evidence that adolescence-onset tobacco, alcohol, and cannabis use are more predictive of dependence problems than adult-onset use [39]. Previous studies show early-onset tobacco and alcohol use are associated with increased risk of suicide [40], NSSI [41], and aggression [42], while cannabis use is associated with increased suicide attempts [43] and NSSI [44]. Our results suggest the summation of risk factors associated with these harming behaviors.

Third, the results suggest dual-harming adolescents may be at increased risk for developing broader psychopathology, including (subthreshold) personality disorders, according to the DSM-5 criteria. In this study dual-harmers appeared to suffer more difficulties in affect regulation and interpersonal functioning, combined with experiencing more unfavorable parenting practices. Since early clinical signs of psychopathology are non-specific, overlapping, and non-linear [45], ‘clinical staging’ using dual-harm as a clear marker may offer a pragmatic and transdiagnostic approach to identify risk and protective factors for future psychopathology [46]. The risk of transitioning to DSM-5 classified psychiatric disorders in dual-harming adolescents needs further study. The follow-up data from the iBerry Study cohort, currently being collected up to 10 years after baseline, may shed light on these transition risks.

Fourth, dual-harm was associated with harsher parenting practices and parental hostility as experienced by the adolescents, but also confirmed by parental self-report. While the association of parental hostility with other-harming behavior is often reported in previous studies, our study shows that harsh parenting was reported as frequent or even more frequent by dual-harming adolescents. The relationship between aggressive behavior and parenting style is complex and likely bidirectional, with an authoritarian style leading to decreases in self-esteem and rising externalizing behavior [47]. In turn, adults may perceive aggressive behavior by their children as more challenging, which may induce more hostile responses, whereas self-harm may trigger more compassionate or supporting behavior. It is noteworthy that the combination of aggressive behavior toward others and self might be even more challenging, with higher needs, also in the parents, for professional support and specialized mental health care. These findings add to earlier findings that dual-harmers more often grow up in high-risk parental environments of unemployment, criminality, and substance misuse [48].

Finally, we found no evidence for differences in IQ-scores between the no harm, other-harm, self-harm, and dual-harm groups. Richmond-Rakerd et al. did find a marginal lower IQ-score in dual-harmers compared to self-harm only [11]. This (large) study operationalized other-harm as at least two self-reported violent behaviors or one official offense by age 22, i.e. more severe forms of aggression toward others. Furthermore, whereas other-harm was more frequently reported by boys, and self-harm more frequently by girls, dual-harm was not sex-specific. Our results at least indicate that dual-harm may occur across a broad spectrum of intelligence scores and in both sexes, which is in line with clinical experience, and therefore requires attention in general (mental) healthcare settings.

Our study in a large group of non-clinically referred adolescents, with multiple informants and multiple methods of data assessment, also has some limitations. First, at baseline of a long-term cohort study, the cross-sectional data cannot be used to infer causality. Second, we operationalized harm to others and self as either present or absent, rather than taking into account frequency or duration of the behavior. As adolescents’ resilience is high, some harming behaviors may have been a one-time occasion. Future research is needed to differentiate between persistent and temporary problems, and the long-term prediction of continuation, severity, and transition into adult psychopathology. Relatedly, we did not account for different forms of other-harming and self-harming behaviors. For example, some forms of self-harm are considered more aggressive than others (e.g. cutting versus pinching), based on the amount of tissue damage [49]. Likewise, criminal laws may provide a way of ranking violent offending severity considering the severity of the punishments a judge may impose. Third, although we used information from both self-report questionnaires and clinical interviews, we cannot rule out the impact of social desirability which may have led to underreporting. On the other hand, adolescents’ self-report on delinquency, self-harm, and substance use is probably the most sensitive source of information on behavior that parents or other informants may not be aware of.

This study has several implications. First, research on adolescents’ aggressive and violent behavior should consider self-harm and vice versa. These behaviors often co-occur and may be indicative of broader problems in terms of psychopathology, functioning, and negative parenting behaviors. Second, more research is needed into underlying mechanisms and long-term outcomes. Our study stresses the need to specifically assess at risk adolescents on dual-harming behaviors and to increase awareness in general practitioners and youth care professionals. An adolescent that reports both self-harm and other-harm would require more specialized diagnostic assessment, close monitoring on continuation of behavior, and early intervention strategies, including family and parental focused treatments to avoid further harm by inadequate parenting responses. While aggressive and violent behavior may receive the most attention in forensic settings, self-harm may attract more attention in regular clinical settings. However, risk assessment in both settings should carefully assess both other-harming and self-harming behaviors. If dual-harm is present, substance use should be further assessed. In addition to (forensic) mental healthcare, a broad range of settings may benefit from further knowledge on dual-harm, for example schools, police and juvenile justice settings, and social workers and psychologists within and outside mental health settings. Third, while short-term suicidality alone or one-time involvement in a fight at school may be part of typical development, more caution is warranted when both co-occur across different settings and domains. This co-occurrence may be indicative of serious psychopathology. Early detection at schools and referral to specialized care may be warranted and could prevent future problems.

## Supplementary Information

Below is the link to the electronic supplementary material.

Supplementary file1 (DOCX 70 KB)
