# Supplementary material for: Dual-harm in adolescence and associated clinical and parenting factors
Source: Soc Psychiatry Psychiatr Epidemiol. 2022 Mar 29;57(8):1615–26. doi: 10.1007/s00127-022-02258-2 (PMC9288948; doi:10.1007/s00127-022-02258-2)
Supplement: Supplementary file 1 — Supplementary file1 (DOCX 70 KB) [file 127_2022_2258_MOESM1_ESM.docx]

**Supplementary Information**

| **Table S1**  *Unadjusted Results ANCOVA Post-Hoc Group Comparisons by Harm Group, with No Harm as Reference Group* | | | | | | | | | | | | |
| --- | --- | --- | --- | --- | --- | --- | --- | --- | --- | --- | --- | --- |
|  | **No Harm** | | **Other-Harm** | | **Self-Harm** | | **Dual-Harm** | | **Between subject effects**^a^ | | | |
| **Outcome** | **Est. Mean** | **SE** | **Mean diff.** | **SE** | **Mean diff.** | **SE** | **Mean diff.** | **SE** | **F_3,1018_** | ***p*** | **η_p_^2^** | **Effects** |
| **Clinical factors** |  |  |  |  |  |  |  |  |  |  |  |  |
| Intelligence scores | 98.45 | 0.68 | -2.67 | 1.11 | -0.45 | 1.24 | -1.73 | 1.24 | 2.18 | .089 | - | N,O,S,D |
| Functioning - adolescent rated | 33.13 | 0.27 | -1.63 | 0.51 | -5.05 | 0.63 | -6.29 | 0.52 | 78.58 | <.001 | 0.19 | N>O>S,D |
| Functioning - parent rated | 32.19 | 0.42 | -2.31 | 0.84 | -3.48 | 0.73 | -5.80 | 0.79 | 42.13 | <.001 | 0.11 | N>O,S>D |
| Functioning - clinician rated | 31.83 | 0.32 | -1.82 | 0.57 | -4.78 | 0.47 | -5.65 | 0.53 | 73.39 | <.001 | 0.18 | N>O>S,D |
| Total emotional and behavioral problems | 22.24 | 0.98 | +8.11 | 1.67 | +10.96 | 1.98 | +18.11 | 1.95 | 38.32 | <.001 | 0.10 | N>O,S>D |
|  |  |  |  |  |  |  |  |  |  |  |  |  |
| **Parenting factors** |  |  |  |  |  |  |  |  |  |  |  |  |
| Parental hostility | 0.67 | 0.06 | +0.08 | 0.10 | +0.00 | 0.10 | +0.32 | 0.10 | 4.63 | .003 | 0.01 | N,O,S<D |
| Harsh parenting - adolescent rated | 1.36 | 0.09 | +0.60 | 0.16 | +0.66 | 0.18 | +1.13 | 0.18 | 17.09 | <.001 | 0.05 | N<O<D;N<S |
| Harsh parenting - parent rated | 0.75 | 0.08 | +0.41 | 0.15 | +0.18 | 0.13 | +0.79 | 0.15 | 15.50 | <.001 | 0.04 | N<O,D;S<D |
| *Note.* Abbreviations: D, Dual-Harm; N, No Harm; O, Other-Harm; S, Self-Harm.  ^a^ Median statistic over pooled datasets. | | | | | | | | | | | | |

| **Table S2**  *Unadjusted Logistic Regression Results for Harm Groups on Substance Use* | | | | | | | | | | | |  |
| --- | --- | --- | --- | --- | --- | --- | --- | --- | --- | --- | --- | --- |
|  | **Harm group (reference = No Harm)** | | | | | | | | | | |  |
|  | **Other-Harm** | | | **Self-Harm** | | | | **Dual-Harm** | | | **Block 2 test^a^** | |
| **Outcome** | **OR** | **95%CI** | ***p*** | **OR** | **95%CI** | ***p*** | **OR** | | **(95%CI)** | ***p*** | ***χ*^2^_3_** | ***p*** |
| Smoking | 3.87 | (2.42 – 6.19) | <.001 | 3.19 | (1.90 – 5.34) | <.001 | 7.48 | | (4.66 – 12.00) | <.001 | 82.01 | <.001 |
| Alcohol | 2.13 | (1.54 – 2.94) | <.001 | 1.59 | (1.12 – 2.28) | .010 | 3.18 | | (2.20 – 4.60) | <.001 | 48.01 | <.001 |
| Illicit Drugs | 4.01 | (2.39 – 6.72) | <.001 | 2.49 | (1.37 – 4.53) | .003 | 6.23 | | (3.69 – 10.51) | <.001 | 58.03 | <.001 |
| ^a^ Median statistic over pooled datasets. | | | | | | | | | | | |  |

| **Table S3**  *Aggressive Behavior Only Results ANCOVA Post-Hoc Group Comparisons by Harm Group, with No Harm as Reference Group* | | | | | | | | | | | | |
| --- | --- | --- | --- | --- | --- | --- | --- | --- | --- | --- | --- | --- |
|  | **No Harm** | | **AB Only** | | **Self-Harm** | | **Dual-Harm** | | **Between subject effects^c^** | | | |
| **Outcome** | **Est. Mean** | **SE** | **Mean diff.** | **SE** | **Mean diff.** | **SE** | **Mean diff.** | **SE** | **F_3,1011_** | ***p*** | **η_p_^2^** | **Effects** |
| **Clinical factors** |  |  |  |  |  |  |  |  |  |  |  |  |
| Intelligence scores^a^ | 97.88 | 0.59 | -2.26 | 1.43 | 0.37 | 1.05 | -2.43 | 1.46 | 2.13 | .095 | - | N,AB,S,D |
| Functioning - adolescent rated^b^ | 32.84 | 0.23 | -1.87 | 0.61 | -4.64 | 0.57 | -6.83 | 0.83 | 71.75 | <.001 | 0.18 | N>AB>S,D |
| Functioning - parent rated^b^ | 31.75 | 0.37 | -2.09 | 1.09 | -3.20 | 0.59 | -6.55 | 1.09 | 40.76 | <.001 | 0.11 | N>AB,S>D |
| Functioning - clinician rated^a^ | 31.45 | 0.28 | -1.84 | 0.70 | -3.80 | 0.43 | -6.80 | 0.77 | 70.61 | <.001 | 0.17 | N>AB>S>D |
| Total emotional and behavioral problems^b^ | 24.14 | 0.87 | +6.75 | 2.19 | +9.29 | 1.70 | +18.91 | 2.87 | 32.46 | <.001 | 0.09 | N<AB,S<D |
|  |  |  |  |  |  |  |  |  |  |  |  |  |
| **Parenting factors** |  |  |  |  |  |  |  |  |  |  |  |  |
| Parental hostility^b^ | 0.69 | 0.05 | +0.11 | 0.11 | +0.03 | 0.08 | +0.35 | 0.14 | 15.79 | <.001 | 0.05 | N,AB,S,D |
| Harsh parenting - adolescent rated^b^ | 1.46 | 0.09 | +0.59 | 0.21 | +0.59 | 0.16 | +1.26 | 0.24 | 15.79 | <.001 | 0.05 | N<S,AB<D |
| Harsh parenting - parent rated^b^ | 0.84 | 0.06 | +0.36 | 0.16 | +0.18 | 0.11 | +0.87 | 0.25 | 12.51 | <.001 | 0.04 | N<AB,D;S<D |
| *Note.* Abbreviations: AB, Aggressive Behavior; D, Dual-Harm; N, No Harm; S, Self-Harm.  ^a^ Controlling for adolescents' age and sex and parental age and sex; F_3,1014_.  ^b^ Controlling for adolescents' age and sex and parental age, sex, and education level.  ^c^ Median statistic over pooled datasets. | | | | | | | | | | | | |

| **Table S4**  *Aggressive Behavior Only Logistic Regression Results for Harm Groups on Substance Use* | | | | | | | | | | | |  |
| --- | --- | --- | --- | --- | --- | --- | --- | --- | --- | --- | --- | --- |
|  | **Harm group (reference = No Harm)** | | | | | | | | | | |  |
|  | **Aggressive Behavior Only** | | | **Self-Harm** | | | | **Dual-Harm** | | | **Block 2 test^b^** | |
| **Outcome** | **OR** | **95%CI** | ***p*** | **OR** | **95%CI** | ***p*** | **OR** | | **(95%CI)** | ***p*** | ***χ*^2^_3_** | ***p*** |
| Smoking^a^ | 4.84 | (2.85 – 8.19) | <.001 | 3.19 | (2.06 – 4.96) | <.001 | 6.65 | | (3.90 – 11.34) | <.001 | 77.75 | <.001 |
| Alcohol^a^ | 1.91 | (1.25 – 2.92) | .003 | 1.71 | (1.23 – 2.36) | .001 | 2.89 | | (1.76 – 4.73) | <.001 | 29.87 | <.001 |
| Illicit Drugs^a^ | 3.56 | (2.02 – 6.26) | <.001 | 2.28 | (1.39 – 3.75) | .001 | 5.79 | | (3.35 – 10.02) | <.001 | 47.76 | <.001 |
| ^a^ Controlling for adolescents' age and sex and parental age, sex, and education level.  ^b^ Median statistic over pooled datasets. | | | | | | | | | | | |  |

| **Table S5**  *Suicidality Only Results ANCOVA Post-Hoc Group Comparisons by Harm Group, with No Harm as Reference Group* | | | | | | | | | | | | |
| --- | --- | --- | --- | --- | --- | --- | --- | --- | --- | --- | --- | --- |
|  | **No Harm** | | **Other-Harm** | | **Suicidality Only** | | **Dual-Harm** | | **Between subject effects^c^** | | | |
| **Outcome** | **Est. Mean** | **SE** | **Mean diff.** | **SE** | **Mean diff.** | **SE** | **Mean diff.** | **SE** | **F_3,1011_** | ***p*** | **η_p_^2^** | **Effects** |
| **Clinical factors** |  |  |  |  |  |  |  |  |  |  |  |  |
| Intelligence scores^a^ | 98.32 | 0.59 | -2.71 | 0.95 | +0.79 | 1.83 | 0.07 | 1.76 | 3.11 | .025 | 0.01 | N>O;N,S,D |
| Functioning - adolescent rated^b^ | 32.56 | 0.25 | -2.40 | 0.42 | -6.73 | 0.91 | -8.33 | 0.86 | 69.34 | <.001 | 0.17 | N>O>S,D |
| Functioning - parent rated^b^ | 31.68 | 0.35 | -2.59 | 0.65 | -4.12 | 1.20 | -7.62 | 1.07 | 41.10 | <.001 | 0.11 | N>O,S>D |
| Functioning - clinician rated^a^ | 31.24 | 0.30 | -2.14 | 0.45 | -6.69 | 0.76 | -7.70 | 0.81 | 74.58 | <.001 | 0.18 | N>O>S,D |
| Total emotional and behavioral problems^b^ | 23.69 | 0.88 | +8.60 | 1.54 | +14.27 | 2.84 | +25.49 | 2.65 | 41.55 | <.001 | 0.11 | N<O,S<D |
|  |  |  |  |  |  |  |  |  |  |  |  |  |
| **Parenting factors** |  |  |  |  |  |  |  |  |  |  |  |  |
| Parental hostility^b^ | 0.68 | 0.05 | +0.09 | 0.08 | -0.02 | 0.14 | +0.57 | 0.14 | 6.69 | <.001 | 0.02 | N,O,S<D |
| Harsh parenting - adolescent rated^b^ | 1.46 | 0.08 | +0.56 | 0.15 | +1.05 | 0.27 | +1.45 | 0.25 | 18.19 | <.001 | 0.05 | N<O<D;N<S |
| Harsh parenting - parent rated^b^ | 0.81 | 0.07 | +0.43 | 0.15 | +0.05 | 0.19 | +0.87 | 0.18 | 13.28 | <.001 | 0.04 | N<O<D;S<D |
| *Note.* Abbreviations: D, Dual-Harm; N, No Harm; O, Other-Harm S, Suicidality.  ^a^ Controlling for adolescents' age and sex and parental age and sex; F_3,1014_.  ^b^ Controlling for adolescents' age and sex and parental age, sex, and education level.  ^c^ Median statistic over pooled datasets. | | | | | | | | | | | | |

| **Table S6**  *Suicidality Only Logistic Regression Results for Harm Groups on Substance Use* | | | | | | | | | | | |  |
| --- | --- | --- | --- | --- | --- | --- | --- | --- | --- | --- | --- | --- |
|  | **Harm group (reference = No Harm)** | | | | | | | | | | |  |
|  | **Other-Harm** | | | **Suicidality Only** | | | | **Dual-Harm** | | | **Block 2 test^b^** | |
| **Outcome** | **OR** | **95%CI** | ***p*** | **OR** | **95%CI** | ***p*** | **OR** | | **(95%CI)** | ***p*** | ***χ*^2^_3_** | ***p*** |
| Smoking^a^ | 4.55 | (3.04 – 6.80) | <.001 | 3.71 | (1.95– 7.05) | <.001 | 6.65 | | (3.64 – 12.15) | <.001 | 77.78 | <.001 |
| Alcohol^a^ | 2.42 | (1.79 – 3.28) | <.001 | 1.33 | (0.74 – 2.37) | .342 | 3.40 | | (1.92 – 6.03) | <.001 | 44.40 | <.001 |
| Illicit Drugs^a^ | 3.99 | (2.55 – 6.25) | <.001 | 1.95 | (0.87 – 4.34) | .103 | 6.21 | | (3.17 – 12.18) | <.001 | 51.18 | <.001 |
| ^a^ Controlling for adolescents' age and sex and parental age, sex, and education level.  ^b^ Median statistic over pooled datasets. | | | | | | | | | | | |  |
